# A Comprehensive Molecular Modeling Study of Phenyltriazolinone Derivatives as Protoporphyrinogen Oxidase (PPO) Inhibitors

**DOI:** 10.1002/cbdv.71268

**Published:** 2026-04-30

**Authors:** Adriana C. de Faria, Antônio Pedro L. Mesquita, Elaine F. F. da Cunha, Matheus P. Freitas

**Affiliations:** ^1^ Department of Chemistry Institute of Natural Sciences Federal University of Lavras Lavras Minas Gerais Brazil

**Keywords:** herbicide, MIA‐QSAR, PBSA, phenyltriazolinone, protoporphyrinogen oxidase IX

## Abstract

Herbicides that inhibit protoporphyrinogen IX oxidase (PPO) constitute an important class of highly effective agrochemicals with broad applications. In this work, an integrated computational strategy was employed to investigate the PPO inhibitory activity of a series of phenyltriazolinone herbicides containing five‐membered heterocyclic rings. The approach combined quantitative structure–activity relationship (QSAR) modeling, molecular docking, molecular dynamics simulations, and Poisson–Boltzmann surface area (PBSA) calculations. Recently synthesized compounds from this class were designed to modify the chemical structures of known PPO inhibitors and enhance their herbicidal performance. QSAR modeling of the available bioactivity data enabled the proposal of new derivatives, among which compound **P7** exhibited a predicted pIC_50_ value of 7.11. Two additional candidates, **P4** and **P5**, were also predicted to display higher herbicidal activity than the reference compound sulfentrazone. These findings were further supported by molecular docking and molecular dynamics analyses, which confirmed favorable binding modes and stable interactions with the PPO enzyme. Binding affinities were additionally evaluated through Gibbs free energy calculations using the PBSA method. Finally, feasible synthetic routes were proposed for the most promising compounds, supporting their potential development as next‐generation PPO inhibitors.

## Graphical abstract

An integrated computational strategy (QSAR, docking, molecular dynamics, and PBSA) identified novel phenyltriazolinone‐based PPO inhibitors with enhanced predicted activity. Compound **P7** emerged as the most promising candidate, while **P4** and **P5** also outperformed sulfentrazone. These findings highlight the potential of rational design for developing next‐generation herbicides.

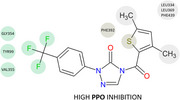

## 1 Introduction

Modern agriculture faces the challenge of maintaining high productivity levels while reducing the environmental impacts caused by excessive use of chemical pesticides. In this context, the enzyme protoporphyrinogen IX oxidase (PPO, E.C. 1.3.3.4) stands out as one of the main molecular targets of selective herbicides. This enzyme is the last step in the biosynthesis of chlorophyll and heme [1] and plays a crucial role in transforming protoporphyrinogen IX into protoporphyrin IX, using FAD (flavin adenine dinucleotide) and molecular oxygen as cofactors. This process results in the release of hydrogen peroxide [2]. In plants, protoporphyrin IX is essential for chlorophyll production, which is crucial for photosynthesis. When this process is disrupted, a toxic accumulation of protoporphyrin IX occurs in the cytoplasm. Under light exposure, this substance generates reactive oxygen species, causing chlorosis, desiccation, and cell death, which results in visible symptoms in affected plants [3]. Herbicides that inhibit PPO, such as diphenylethers, triazolinones, and benzothiazoles, have been widely used in weed control. However, the continuous use of these substances has led to the emergence of resistance, highlighting the need to develop more selective and sustainable compounds [4, 5, 6]. Therefore, the search for herbicides that inhibit PPO remains an effective strategy for weed control, helping to protect crops, and reduce environmental damage.

Some structural characteristics of PPO herbicides can increase foliar absorption and translocation in plants, such as the presence of hydrophobic side chains, electron‐withdrawing groups, among others. The different hydrophobic portions in their structures are responsible for making them more easily absorbed by the foliage and transferred to other parts of the plant, which is related to their improved binding affinity with plant PPOs [3]. The presence of electron‐withdrawing groups, such as C─F bonds, is responsible for increasing the lipophilicity of the molecules, thereby improving their bioavailability, which can be used to protect or inhibit metabolism in plants [7]. Therefore, physicochemical parameters such as log *P* (octanol/water partition coefficient) are essential for evaluating the lipophilicity, solubility, and permeability of compounds, directly influencing their efficacy and selectivity [8].

**FIGURE 1:**
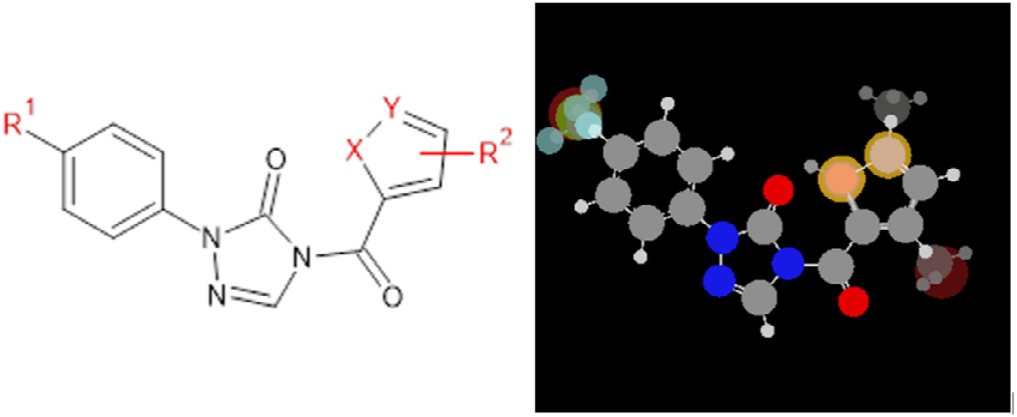
The phenyltriazolinones derivatives studied herein and the superimposition of the 33 compounds as colored ball‐and‐stick schemes (H = white, C = grey, N = blue, O = red, F = electric blue, S = yellow, Cl = green, and Br = dark red). The colors are in the RGB scale (varying from 0 to 765‐pixel values), which were replaced with the corresponding electronegativity (ε) and van der Waals radius (r_vdW_).

Considering the potential development of resistance to known herbicides, a series of herbicides from the phenyltriazolinone class containing five‐membered heterocyclic rings [7] have been synthesized and tested against PPO to demonstrate better performance of compounds that feature the C─F bond, compared to sulfentrazone (Figure 1). In this sense, modifications to the chemical structure of these compounds, guided by molecular modeling, such as the introduction of fluorine into the molecular structure, are useful for developing lead compounds, which may generate new agrochemical candidates with improved activities compared to existing compounds.

To enhance the performance of agrochemicals and also address resistance issues, it is important to identify and understand how changes in the chemical structure of known compounds can lead to the development of new bioactive molecules. For this, modeling strategies, such as quantitative structure‐activity relationship (QSAR), docking, and molecular dynamics, are used. These approaches, which involve analyzing both the ligand and the receptor, follow the rigorous validation protocols found in the literature. These protocols ensure the reliability, predictive power, and robustness of the models, as well as provide information on possible modes of interaction between a ligand molecule and its biological target, usually an enzyme, through the molecular docking technique [9, 10, 11]. Together, these methods are capable of predicting and explaining the biological activities of new compounds being proposed.

Most of the amino acids that form a protein interact through long‐range forces, primarily electrostatic in nature. The electric fields produced by proteins can extend up to about 10–15 Å, depending on factors such as temperature, solvent, and protein charge. Therefore, to understand how biomolecules attract or repel each other, it is important to comprehend the laws of electrostatic physics that explain these interactions. In this work, an introduction will be made to the use of the Poisson‐Boltzmann equation − PBSA (Poisson–Boltzmann Surface Area, Generalized‐Born surface area) [12] to investigate the energetic feasibility of the protein‐ligand complex, understanding how intermolecular interactions influence the ∆G value calculated by PBSA. Thus, the integration of MIA‐QSAR, PBSA, and log *P* enables a more robust and predictive approach in the virtual screening of phenyltriazolinones with herbicidal activities.

## 2 Materials and Methods

### 2.1 QSAR Analysis

A series of related compounds, consisting of 33 phenyltriazolinone derivatives containing five‐membered heterocyclic rings, were obtained from the literature [7]. The inhibition constants for Nicotiana tabacum PPO (expressed in terms of IC_50_, that is, the concentration of inhibitor required for 50% of the maximum effect against Nicotiana tabacum PPO, in mg L^−1^) were reported. The herbicidal activity data were converted into pIC_50_ (‐log IC_50_ in mol L^−1^) and used in MIA‐QSAR modeling. The compound dataset and their respective biological values used in the MIA‐QSAR analyses are presented in Table 1.

**TABLE 1:** Phenyltriazolinone derivatives containing five‐membered heterocyclic rings and their respective PPO inhibitory activity (IC_50_ in mg L^−1^) (pIC_50_, IC_50_ in mol L^−1^).

Compounds	X	Y	*R* ^1^	*R* ^2^	IC_50_ (mg L^−1^)	pIC_50_ (mol L^−1^)
**1** ^b^	S	CH	H	H	1.404	5.29
**2** ^b^	S	CH	H	3‐CH_3_	1.315	5.34
**3**	S	CH	H	3‐Br	1.658	5.32
**4**	S	C	H	5‐CH_3_	1.173	5.39
**5**	CH	S	H	H	1.818	5.17
**6** ^b^	O	CH	H	H	2.125	5.08
**7** ^a^	S	CH	Cl	H	0.403	5.88
**8**	S	CH	Cl	3‐CH_3_	0.362	5.95
**9** ^a^	S	CH	Cl	3‐Br	0.524	5.87
**10** ^a^	S	C	Cl	5‐CH_3_	0.243	6.12
**11** ^a^	CH	S	Cl	H	0.672	5.66
**12**	O	CH	Cl	H	0.691	5.62
**13**	S	CH	CH_3_	H	2.638	5.03
**14** ^b^	S	CH	CH_3_	3‐CH_3_	1.987	5.18
**15** ^a^	CH	S	CH_3_	H	3.042	4.97
**16** ^b^	O	CH	CH_3_	H	3.412	4.90
**17**	S	CH	CF_3_	H	0.058	6.77
**18**	S	CH	CF_3_	3‐CH_3_	0.044	6.90
**19**	S	CH	CF_3_	3‐Br	0.068	6.79
**20**	S	C	CF_3_	5‐CH_3_	0.033	7.03
**21**	O	CH	CF_3_	H	0.090	6.56
**22**	S	CH	Br	H	0.815	5.63
**23** ^a^	S	CH	Br	3‐CH_3_	0.785	5.67
**24**	S	CH	Br	3‐Br	0.968	5.65
**25**	S	C	Br	5‐CH_3_	0.654	5.75
**26**	CH	S	Br	H	1.118	5.50
**27** ^a^	O	CH	Br	H	1.241	5.43
**28**	S	CH	F	H	0.136	6.33
**29**	S	CH	F	3‐CH_3_	0.106	6.46
**30** ^b^	S	CH	F	3‐Br	0.167	6.34
**31** ^b^	S	C	F	5‐CH_3_	0.093	6.51
**32** ^b^	CH	S	F	H	0.179	6.21
**33** ^a^	O	CH	F	H	0.245	6.05
**Sulfentrazone** ^c^					**0.078**	**6.70**

^a^ Test set compounds selected with Kennard‐Stone sampling for the Pauling electronegativity model (ε).

^b^ Test set compounds selected with Kennard‐Stone sampling for the van der Waals radius (r_vdW_) model.

^c^ Reference compound.

For the MIA‐QSAR modeling, the following procedure was carried out: the 33 chemical structures were sketched as ball‐and‐stick models using the GaussView program [13], where the balls (atoms) were scaled proportionally to their respective van der Waals radii and colored according to standard preferences using the RGB scale (ranging from 0 for black to 765 for white). All structures were aligned so that the common core remained in the same position, in terms of pixels (Figure 1), across all of them, ensuring perfect overlap of the compounds at common points. The images were saved as bitmaps with dimensions of 300 × 348 pixels. The RGB pixel values were renumbered to correspond to chemical properties, such as Pauling's electronegativity or the van der Waals radius values of the corresponding atoms.

Thus, each 2D chemical structure, represented as 300 × 348 data points, was unfolded into a vector of 1 × 104,400 rows and subsequently grouped to form a matrix X with dimensions 33 × 104,400. The applicability domain and the presence of outliers were assessed using William's plots (leverage and Student's residual analysis) [14]. Subsequently, the matrix was divided into training (75%) and test (25%) sets through Kennard‐Stone sampling [15]. The training set was used for calibration (regression with pIC_50_ values via PLS), leave‐one‐out cross‐validation, and y‐randomization tests, while the test set was used for external validation. The quality of the MIA‐QSAR model was evaluated based on well‐known calibration and validation metrics; that is, root mean square errors (RMSE), *r* ^2^ (values ≥ 0.6 are acceptable) [16], q^2^ [17], r^2^_pred_ [11], ^c^r^2^_p_ (values ≥ 0.5 are acceptable) [18], r^2^_m_ [19], CCC parameters [20], MAE [21], and Q^2^_F1_ and Q^2^_F2_ [20].

The stability of the models was also evaluated by a bootstrapping procedure, in which several test sets were randomly selected (10 times), and the calibration and validation steps were subsequently performed for each cycle (average statistical values were collected). This procedure is particularly necessary for small datasets to avoid the risk of atypical behavior, for example, due to random correlation, lack of robustness to resampling and cross‐validation [22].

The effect of substituents was analyzed using MIA plots, which are contour maps of scores (VIP) and PLS regression coefficients (**b**) [23]. Overall, these plots indicate, as heat maps, how much each molecular descriptor contributes to the variance in pIC_50_ (VIP) and how they affect these values ​​by decreasing or increasing pIC_50_, (**b**). This procedure was performed using Chemoface [24].

### 2.2 Docking Studies

In order to elucidate the binding affinity of herbicides derived from phenyltriazolinones with the PPO enzyme (EC 1.3.3.4), molecular docking studies were carried out. The crystal structure of PPO complexed with the inhibitor INH (4‐bromo‐3‐(5’‐carboxy‐4’‐chloro‐2’‐fluorophenyl)‐1‐methyl‐5‐trifluoromethylpyrazole) and the cofactor FAD (flavin adenine dinucleotide), at a resolution of 2.90 Å, was obtained from the Protein Data Bank (PDB ID: 1SEZ) [25].

The ligands were prepared and optimized using the BIOVIA Discovery Studio software (https://discover.3ds.com) and the docking simulations were carried out with the assistance of the Molegro Virtual Docker (MVD) software [26]. This software calculates the interaction energy to identify the most likely conformation between the protein and the ligand.

MVD is widely recognized for its efficient performance, combining speed, and accuracy in docking analyses. The software employs search and optimization algorithms that enable effective exploration of the conformational space of molecules. It offers detailed configuration options for various docking parameters, such as defining specific regions of the protein (cavities) and customizing scoring criteria, providing flexibility to adapt the program to specific experimental needs [26].

The MolDock Score [GRID] function uses a hybrid search algorithm that integrates differential evolution optimization with a cavity prediction algorithm during the search process. This integrated approach allows for rapid and accurate identification of potential binding modes and poses. The grid‐based scoring functions pre‐calculate potential energy values on a uniformly spaced cubic grid to accelerate calculations. The energy potential is evaluated using trilinear interpolation between specific grid points. To define the binding site, a grid with a resolution of 0.30 Å and a radius of 15 Å around the inhibitor were applied.

The MolDock SE (Simplex Evolution) search algorithm was employed, with a maximum limit of 100 poses to be generated and 1500 iterations for each pose. In the molecular docking procedure, flexibility was considered for the side chains of amino acids. Potential binding sites were identified using the integrated cavity detection algorithm. A grid covering the protein was established, positioning a sphere at each grid point, some of them overlapping the areas defined by the van der Waals radii (r_vdW_) of the protein atoms. The cavities detected by the algorithm were then analyzed using the guided differential evolution search algorithm, which directed the investigation to specific areas of the cavities during the docking simulation.

Thus, by adopting these computational approaches, it becomes possible to explore the interactions between ligands and receptors, which can provide valuable information about the binding patterns and possible interaction sites of phenyltriazolinone derivatives with the PPO enzyme.

### 2.3 Molecular Dynamics, MM‐PBSA Calculations and Toxicological Predictions

For the molecular dynamics simulations, the ligand candidates resulting from the QSAR step were prepared using ACPYPE [27], which is included in the AmberTools23 package [28]. All simulation steps were performed using GROMACS (2021.4) [29]. The poses of the ligands and the FAD coenzyme were prepared at a neutral protonation state (pH = 7.0) using Discovery Studio, and their topology files were generated with the OPLS‐AA force field [30] via ACPYPE. The MKTOP.pl script [31] was used to correct and refine the AMBER‐recognized atom types. The PPO protein (PDB‐ID: 1SEZ) was protonated at pH 7.0 using the PDB2PQR server [32] for the AMBER force field, based on p*K*a prediction calculations for each amino acid residue performed by PropKA. Following this process, the protein's topology file was generated using GROMACS with the OPLS‐AA force field and the TIP4P water model [33].

The topologies for the protein‐ligand‐FAD complexes were built by merging the topology files of each component. The complexes were centered in a cubic solvation box with dimensions extending 1.5 nm beyond their edges; 62,305 water molecules were added as solvent. To neutralize the system's charge, 4 water molecules were replaced with chloride ions. Minimization steps were performed using the Steepest Descent algorithm [34] with and without position restraints, each for 20,000 steps, followed by an additional LBFGS minimization [35] for 20,000 steps to ensure the systems reached a potential energy minimum. Equilibrations were performed under canonical (NVT) and isothermal‐isobaric (NPT) ensembles. The equilibrations were set to a temperature of 298.15 K and a pressure of 1 bar. The duration was 100 ps, with a time step (dt) of 2 fs. At the end of the equilibration, all systems reached averages close to 298.15 K and a density of 1 kg m^−3^. A solvent relaxation dynamic was performed for 500 ps. Finally, the production (unrestrained) molecular dynamics was run for 200 ns with a time step (dt) of 0.002 ps (2 fs). The Verlet cut‐off scheme [36] and the leap‐frog differential integrator [37] were employed in this stage. RMSD, RMSF, and energy analyses and plots were generated using XMGRACE.

For the MM‐PBSA calculations, the complex trajectories were centered, and periodic boundary conditions (PBC) were removed. To obtain more precise results for configurational entropy, the translational and rotational movements of the complexes were also removed. The calculations were performed using the gmx_MMPBSA (v. 1.6.4) software [38]. The last 50 ns of the simulation were selected for the PBSA calculations, spanning simulation frames 7500 to 10000. Frames were sampled at an interval of 12, totaling 209 frames. The temperature was set to 298.15 K, and the entropy was calculated using the interaction entropy (IE) approximation [39]. The polar solvation free energy was calculated by solving the linear form of the Poisson‐Boltzmann (PB) equation; the nonpolar solvation energy was calculated using the SASA (Solvent‐Accessible Surface Area) model, with the dispersion term based on a surface integration model. The external (water) dielectric constant was set to 80, and the internal (protein) dielectric constant was set to 1. The molecular mechanics energy terms (electrostatic and van der Waals) were obtained from the molecular dynamics simulations.

The toxicity predictions for the QSAR‐designed compounds and the reference molecules were performed using the DeepPK server (https://biosig.lab.uq.edu.au/deeppk/). The following endpoints were evaluated: AMES mutagenesis, avian toxicity, bee toxicity, biodegradation, rat acute toxicity, rat chronic oral toxicity, and Fathead Minnow toxicity.

## 3 Results and Discussion

### 3.1 QSAR Analysis

**FIGURE 2:**
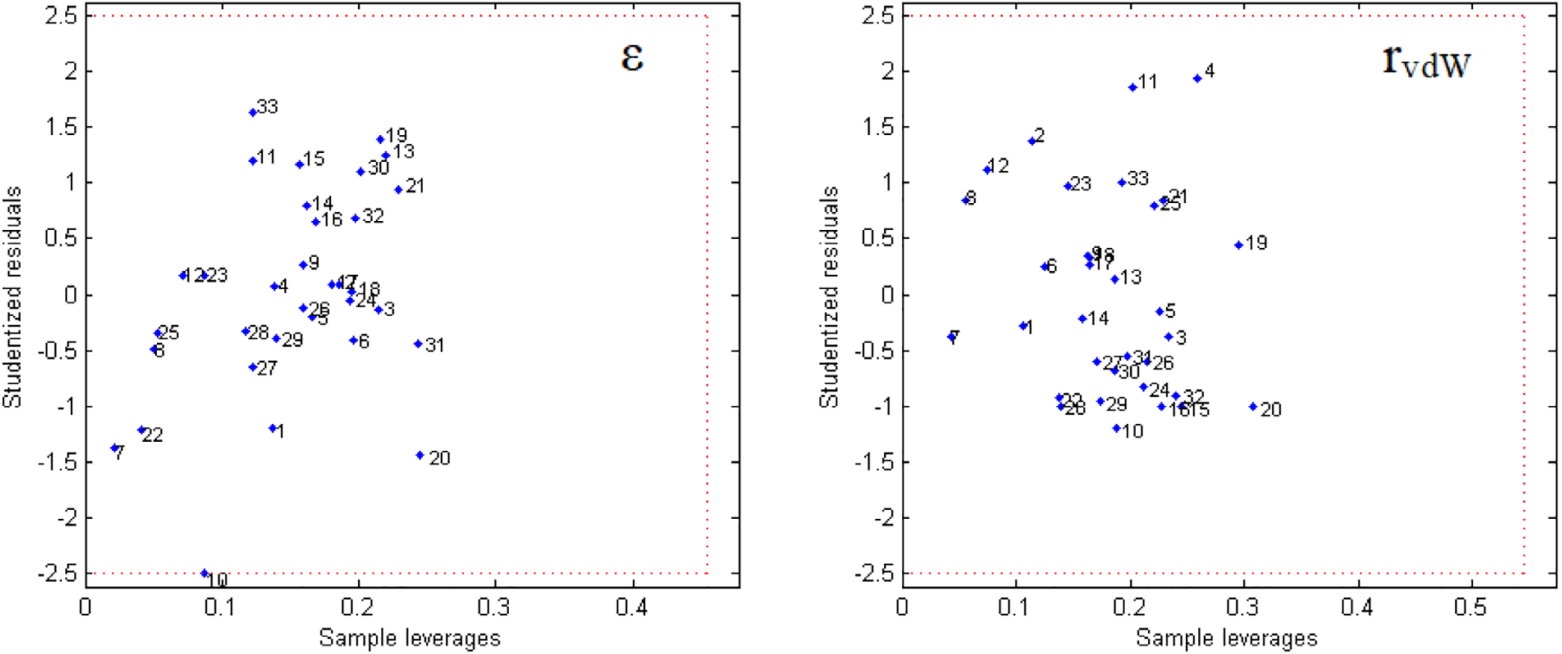
William´s plots used for applicability domain tests.

Before starting the MIA‐QSAR modeling, the 33 compounds in Table 1 were screened to assess their homogeneity and adherence to the chemical space under analysis, using William's plots for outlier detection (Figure 2). Since all compounds fall within the leverage and Student's residual cutoff values, showing no outliers in the evaluated dataset [14], the entire series of molecules was considered in the QSAR studies.

**FIGURE 3:**
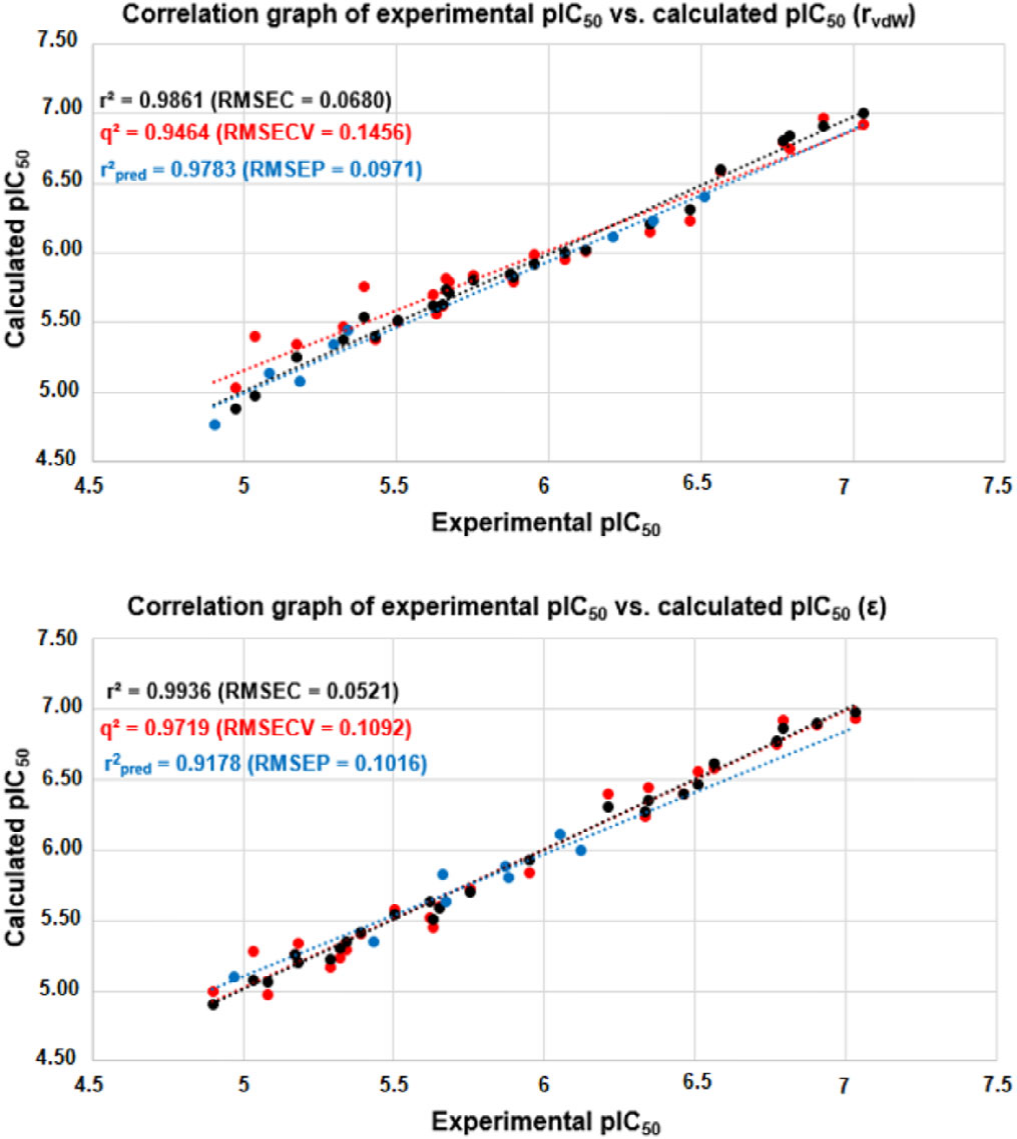
Correlation plots of experimental versus calculated pIC_50_ using MIA‐QSAR based on descriptors related to van der Waals radius (r_vdW_) and Pauling´s electronegativity (ε).

Both models based on MIA descriptors encoding the r_vdW_ and ε properties were not obtained by random correlation, since r^2^ >> r^2^_y‐rand_ (^c^r^2^_p_ ≥ 0.5) and were satisfactorily reliable and predictive (q^2^, r^2^_pred_, mean r^2^_m_ ≥ 0.5, and CCC ≥ 0.85) (Table 2). Additionally, the models were reasonably stable, as the statistical parameters for the bootstrapping experiments (mean for 10 cycles) remained consistently high at each step. However, considering one of the most important parameters for assessing the predictive capacity of a QSAR model [40], the r^2^_pred_ obtained for the molecules in the test set, the r_vdW_‐based model proved to be slightly more powerful than the ε‐based model (Figure 3). This behavior may indicate a more important role for steric effects, rather than electrostatic effects, at the PPO binding site to describe biological activity. Consequently, further discussions on the MIA‐QSAR results will be based on the two models in question, since this difference was 0.0605, a very small difference. Therefore, the MIA‐QSAR model is suitable for predicting the pIC_50_ values ​​of unknown phenyltriazolinone derivatives.

**TABLE 2:** Statistical parameters to assess the quality of MIA‐QSAR models, using Kennard‐Stone (K‐S) sampling to select compounds for the test set and a bootstrapping procedure with 10 cycles of random selection of compounds for the test set.

Parameters	r_vdW_ (K‐S)	ε (K‐S)	r_vdW_ (bootst.)	ε (bootst.)
PLS components	6	5	5.9 ± 0.3	5.1 ± 0.6
RMSEC	0.0680	0.0521	0.0521 ± 0.019	0.0474 ± 0.009
*r* ^2^	0.9861	0.9936	0.9914 ± 0.007	0.9935 ± 0.003
RMSE_y‐rand_	0.4595	0.5255	0.4763 ± 0.021	0.4715 ± 0.019
*r* ^2^_y‐rand_	0.3609	0.3388	0.3643 ± 0.034	0.3765 ± 0.025
^c^ *r* ^2^_p_	0.7852	0.8066	0.7883 ± 0.020	0.7828 ± 0.016
RMSECV	0.1456	0.1092	0.1146 ± 0.048	0.1042 ± 0.020
*q* ^2^	0.9464	0.9719	0.9587 ± 0.037	0.9691 ± 0.011
RMSEP	0.0971	0.1016	0.0978 ± 0.035	0.0892 ± 0.022
*r* ^2^_pred_ *r^2^*_m_ *_(test)_*	0.9783 0.9286	0.9178 0.7870	0.9734 ± 0.020 0.9368 ± 0.035	0.9786 ± 0.008 0.9456 ± 0.045
Avg. *r* ^2^_m_	0.9783	0.8289	0.9205 ± 0.041	0.9347 ± 0.045
Δ*r* ^2^_m_	0.0095	0.0782	0.0232 ± 0.015	0.0195 ± 0.008
CCC	0.9862	0.9535	0.9830 ± 0.011	0.9863 ± 0.006
Q^2^_F1_	0.9787	0.9300	0.9701 ± 0.021	0.9750 ± 0.011
Q^2^_F2_	0.9735	0.9149	0.9661 ± 0.023	0.9727 ± 0.011
MAE	0.0944	0.0885	0.0835 ± 0.034	0.0779 ± 0.018

It is worth noting that previous QSAR studies on different classes of PPO inhibitors have shown acceptable model performance [4, 5, 6, 41], whereas the present study exhibits superior results, particularly in terms of predictive ability. This improvement is not attributable to overfitting, as confirmed by the y‐randomization tests, which demonstrate the robustness of the model. Nevertheless, experimental validation is still required to further support these findings.

**FIGURE 4:**
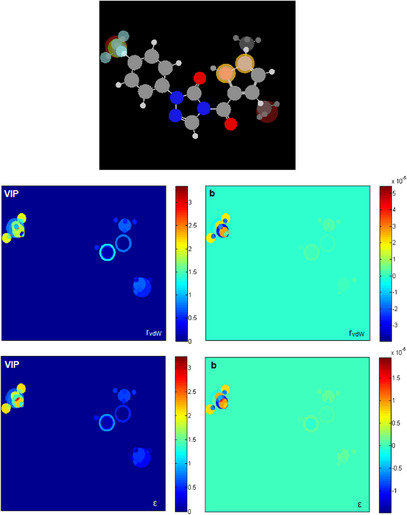
MIA plots of variable importance in projection (**VIP**) and PLS regression coefficients (**b**), and overlaid images used to generate the molecular descriptors of MIAr_vdW_ and MIAε (H = white, C = gray, N = blue, O = red, S = yellow, F = light blue, Cl = green, Br = dark red).

Considering that both QSAR models based on MIA descriptors are suitable for predicting the pIC_50_ values ​​of new derivatives, MIA plots were obtained to analyze how different substituent groups affect these values, and subsequently, to guide the proposition of potential new herbicides (Figure 4). To identify new promising derivatives, compounds with pIC_50_ above 6.90 were focused on, and variable importance projection scores (VIP) MIA plots and PLS regression coefficients (**b**) were analyzed.

The VIP plot shows the contributions of each substituent group to the model, ranging from dark blue (zero contribution) to dark red (large contribution). Graph **b** indicates which substituents favor (red region) or disfavor activity (blue region). Substituents −CF_3_ and −F in R^1^ notably influence pIC_50_ values; in addition, the −CH_3_ group in *R* ^2^ and the −S in X and Y also have a portion of influence on biological activity.

In the VIP plot, it can be observed that the effect of fluorine (−F), trifluoromethyl (−CF_3_), and methyl (−CH_3_) groups at position R^1^ is notable, as well as the presence of a methyl group (−CH_3_) instead of hydrogen (−H) or bromine (−Br) at positions 3 and 5 in *R* ^2^. Secondary effects of sulfur (−S) at positions X and Y are also observed.

The **b** plot indicated that methyl substitution at positions 3 and 5 in *R* ^2^ favors activity (orange region), while −H and −Br decrease the pIC_50_ value (blue region). These findings align with the SAR analysis in the literature [7], which demonstrated that introducing different substituents, including −CH_3_ and −Br, into the thiophene ring, resulted in compounds with varying PPO inhibitory activities. Notably, the introduction of a methyl group (−CH_3_) at positions 3 and 5 of the thiophene ring showed promising results, likely due to the lower steric hindrance, which is more favorable for PPO inhibitory activity. The influence of the −CF_3_ and −F groups in R^1^ is positive, while larger halogens (−Cl and −Br) and methyl (−CH_3_) groups decrease the pIC_50_. The presence of fluorine in R^1^ is beneficial. These findings are consistent with the SAR analysis from the literature [7], which demonstrated that PPO inhibitory activity of the compounds also varied significantly due to the different substituents introduced at the *para* position of the benzene ring (R^1^). The analysis showed that the activity was greater after introducing −CF_3_ into the benzene ring. This could be because the fluorine atom has a small radius and high electronegativity, and the C─F bond formed is much stronger than the C─H bond, thus increasing the structural stability. In general, compounds with electron‐withdrawing groups at the *para* position of the benzene ring exhibited stronger PPO inhibitory activity. Therefore, the greater the electron‐withdrawing ability, the higher the enzymatic inhibitory activity of the compounds.

**SCHEME 1:**
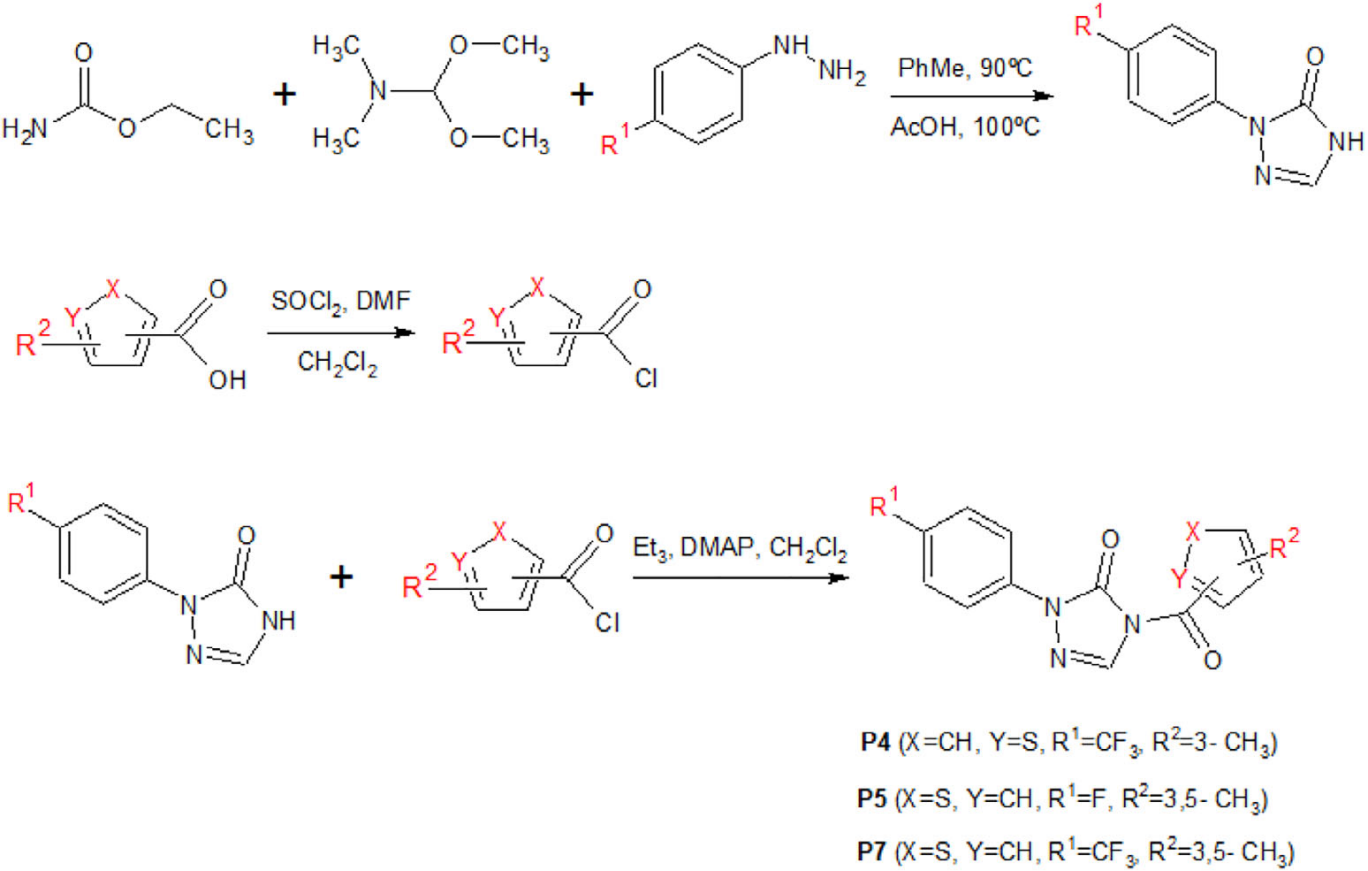
Possible synthetic route for obtaining **P4** and **P7** (R^1^ = −CF3) and **P5** (R^1^ = −F), similar to the procedures described in the literature [7].

Therefore, the combination of the R^1^, *R* ^2^, X, and Y groups is insightful for the development of new promising derivatives for PPO inhibition. Eight compounds (**P1‐P8**) were proposed (Table 3), and their respective pIC_50_ values were estimated using the regression parameters from the MIA‐QSAR model. Synergistic effects of the substituents were observed for one of the proposals, with a calculated pIC_50_ value higher than the experimental value obtained for compound **20** (7.03), and one proposal with a calculated pIC_50_ value higher than the experimental value obtained for the reference compound, sulfentrazone (6.70). Notably, compounds **P4**, **P5**, and **P7** showed pIC_50_ values above 6.50, indicating promising potential for further development through synthetic routes (as suggested in Scheme 1) and subsequent biological assays to validate the computational findings. It is worth noting that **P5** and **P7** differ only in the R^1^ substituent, while *R* ^2^ = 3,5−CH_3_ and X = −S play essential roles in the biological activity, as highlighted by the MIA plots.

**TABLE 3:** Derivatives proposed based on insights from MIA‐plots and the respective calculated pIC_50_ values ​​(IC_50_ in mol.L^−1^, average of the r_vdW_ and ε models).

Proposal	X	Y	*R* ^1^	*R* ^2^	pIC_50_ (mol L^−1^)
**P1**	CH	S	F	3‐CH_3_	6.33
**P2**	S	CH	CH_3_	5‐CH_3_	5.22
**P3**	CH	S	CH_3_	3‐CH_3_	5.11
**P4**	CH	S	CF_3_	3‐CH_3_	**6.88**
**P5**	S	CH	F	3,5‐CH_3_	**6.55**
**P6**	S	CH	CH_3_	3,5‐CH_3_	5.34
**P7**	S	CH	CF_3_	3,5‐CH_3_	**7.11**
**P8**	S	CH	H	3,5‐CH_3_	5.60

**FIGURE 5:**
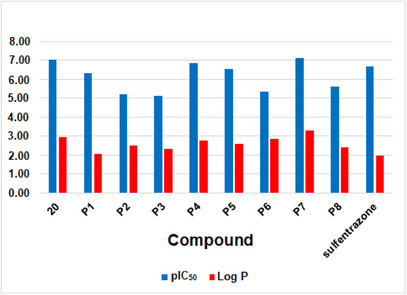
Comparison of calculated pIC_50_ and log *P* (https://molinspiration.com) of the representative compound from the dataset (**20**) and sulfentrazone with the proposed compounds **P1** to **P8**.

Lipophilicity is a parameter widely used to understand bioactivity, especially in terms of cell permeation and leaf absorption. It is usually evaluated by the partition coefficient between octanol and water, known as log *P*. Compounds with high lipophilicity tend to move less in soils rich in organic matter, which reduces the risk of runoff or leaching, although this may raise concerns about bioaccumulation. According to the log *P* calculated on the Molinspiration platform (https://molinspiration.com), the best candidates for PPO inhibitors, such as **P7**, should exhibit higher lipophilicity than most of the active compounds present in the phenyltriazolinone series (Figure 5). In particular, the larger log *P* predicted for **P7** suggests enhanced penetration through the lipid‐rich cuticular layer of plant leaves, which can improve uptake and facilitate access to the target enzyme, potentially increasing herbicidal efficacy. Additionally, its higher lipophilicity may promote stronger interactions within hydrophobic regions of the PPO active site, contributing to improved binding affinity. This clearly demonstrates the advantages of the proposed derivatives compared to known compounds. Thus, the synthesis of these derivatives, as suggested in Scheme 1, along with field testing and biological studies, could be of interest for future research.

### 3.2 Docking Studies

To explore the interactions that drive the inhibitory activity of the compounds against the PPO enzyme, docking studies were performed for sulfentrazone, INH (the native ligand), phenyltriazolinones from the library, as well as the proposed analogs, with a particular emphasis on the most promising candidates. The active site of the PPO analyzed was defined as a region between the FAD cofactor and the substrate‐binding domain. The substrate binding site below the cofactor is a planar cavity formed by some aromatic and aliphatic amino acids, as well as Asn67 and Arg98 (which participate in the localization of the tetrapyrrole ring during the catalytic process of protoporphyrinogen IX). A cavity of 3078.66 Å^3^ was selected.

**FIGURE 6:**
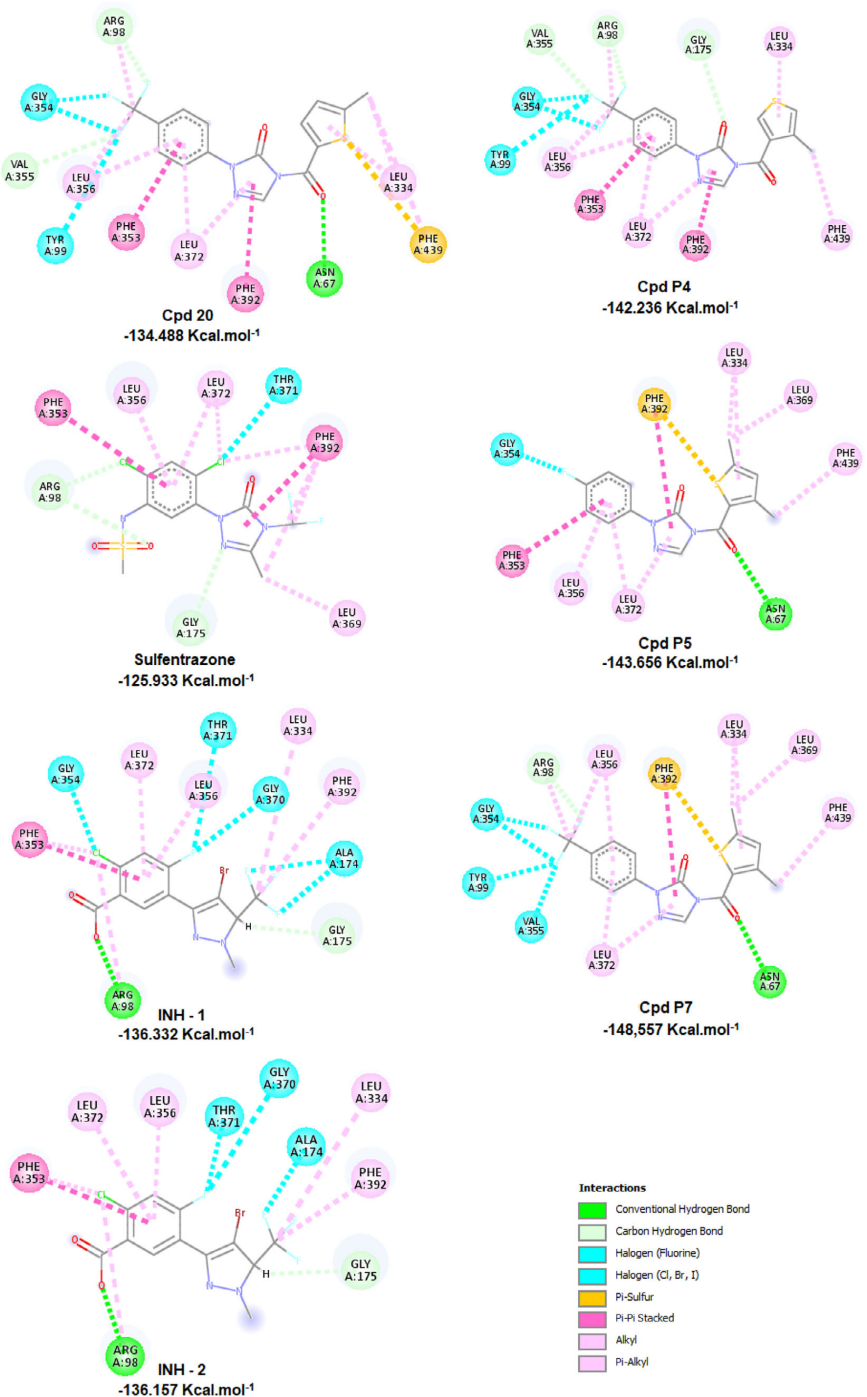
Interactions of promising compounds (**P4**, **P5,** and **P7**) with the PPO enzyme and comparison with compound **20**, sulfentrazone, INH‐1 and INH‐2.

During the docking process, MolDock SE [19] screened a vast number of conformations for each molecule. The first requirement for a useful scoring function is the ability to reproduce the co‐crystallized binding geometry and the orientation of the associated ligand, given a rigid macromolecule state. Thus, the docking studies were confirmed by the repositioning of INH in the cavity and by the overlap of its position with the original crystallized complex. Two initial arrangements were considered: the original one present in the PDB (INH‐1) and the energy‐minimized conformation (INH‐2) RMSDs of 0.38 Å and 0.68 Å were observed for INH‐1 and INH‐2, respectively. The poses were selected according to two criteria: the orientation of INH in the binding site (experimental information) and the pose with the lowest energy. As shown in Figure 6, INH is positioned near the residues Arg98, Ala174, Gly175, Leu334, Phe353, Leu356, Gly370, Thr371, Leu372, and Phe392. Sulfentrazone binds to the active site and mediates several interactions, including carbon‐hydrogen interactions with Arg98 and Gly175, π−π stacking interactions with Phe353 and Phe392, halogen interactions with Thr371, π−alkyl interactions with Phe392, Leu356, and Leu372, and alkyl interactions with Leu369 and Leu372. The binding mode of the most active compound (**20**) shows that the −CF_3_ substituent at the R^1^ position interacts with the amino acid residues Tyr99 and Gly354 through halogen interactions, with Arg98 and Val355 through carbon‐hydrogen interactions, and alkyl interactions with Arg98 and Leu356. The hydrophobic aromatic characteristic in the benzene ring is favorable for π−π stacking interactions with Phe353, π−alkyl interactions with Leu356, the substituent X = S performs π−sulfur type interactions with Phe439, the substituent *R* ^2^ = 5‐CH_3_ forms π−alkyl interactions with Leu334 and Phe439 and alkyl interactions with Leu334. The proposed compound **P4** interacts with PPO through carbon‐hydrogen bonds with Arg98 and Val355, halogen bond interactions with Tyr99, Gly354, and Val355, π‐alkyl interactions with Leu356, and alkyl interactions with Arg98 and Leu356. The proposed compound **P5** interacts with PPO through a conventional carbon‐hydrogen bond with Asn67, halogen bond interaction with Gly354, π−π stacking interactions with Phe353 and Phe392, π−alkyl interactions with Leu334, Leu356, Leu372, Phe439, alkyl interactions with Leu369, and π−sulfur interactions with Phe392. The proposed compound **P7** interacts with PPO through a conventional carbon‐hydrogen bond with Asn67, carbon‐hydrogen bonds with Arg98, halogen bond interaction with Tyr99, Gly354, Val355, π−π stacking interactions with Phe392, π−alkyl interactions with Leu334, Leu372, Phe439, alkyl interactions with Leu334, Leu356, Leu369, and π−sulfur interactions with Phe392.

The proposed compounds **P4**, **P5**, **P6**, and **P7**, with substituents R^1^ = CF_3_, F, CH_3_, and CF_3_, respectively, showed more negative Mol‐Dock Scores than compound **20**, sulfentrazone, and INH. These results support the importance of CF_3_ and F groups for the efficiency of fluorinated compounds.

### 3.3 Molecular Dynamics and MM‐PBSA

**FIGURE 7:**
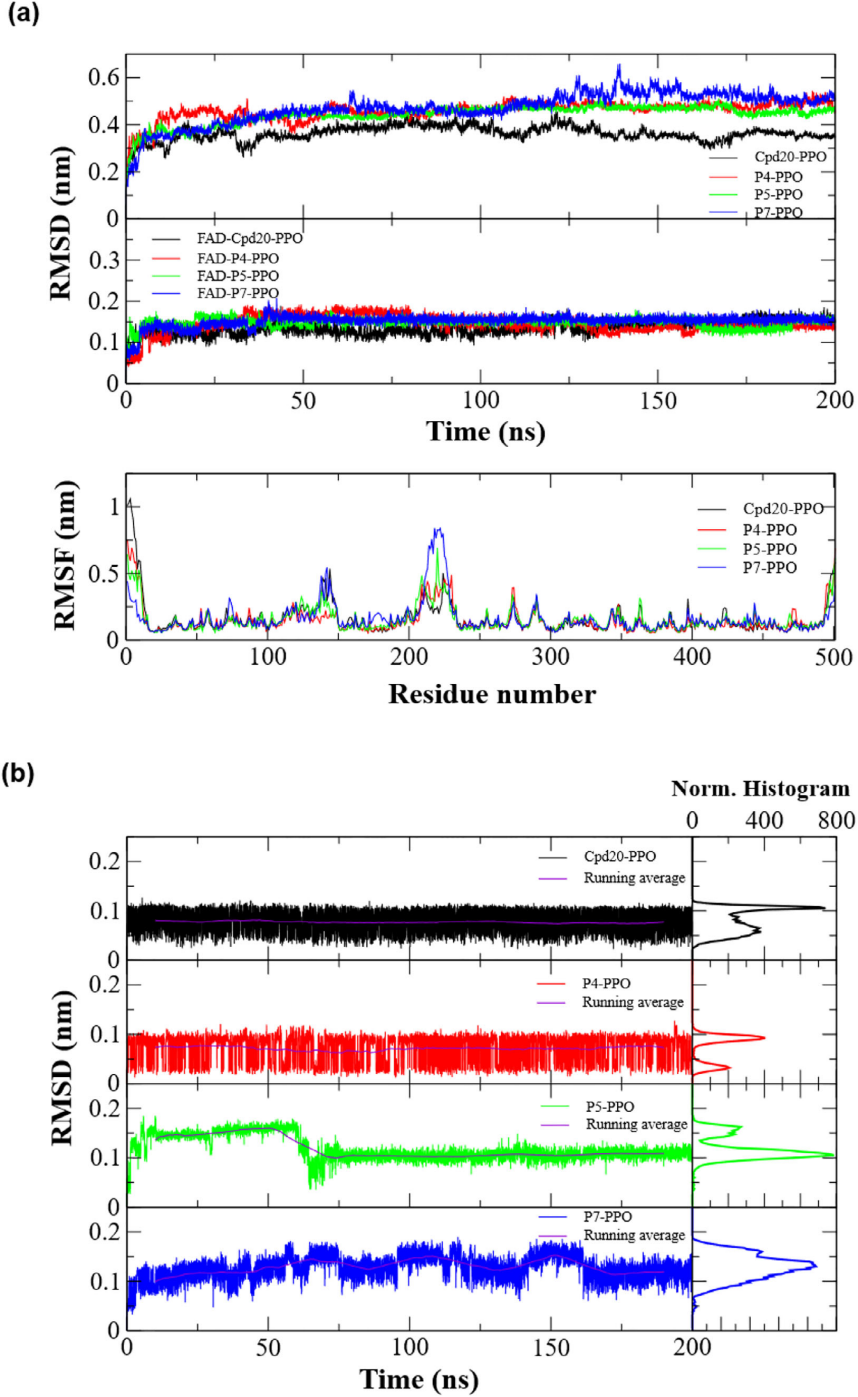
Graphical representation of RMSD and RMSF for the systems subjected to molecular dynamics. (a) RMSD of the protein (PPO) complexes with ligands (**20**, **P4**, **P5**, and **P7**); RMSD of FAD complexed with ligands **20**, **P4**, **P5**, and **P7**; and the RMSF plot of the complexes as a function of the amino acid residue number. (b) RMSD of the ligands complexed with PPO, along with their normalized histograms.

The graphs from the molecular dynamics simulations are presented in Figure 7. The RMSD data for the proteins complexed with the ligands can be found in Figure 7a, referring to the complexes formed with ligands **20**, **P4**, **P5**, and **P7**. The ligand **20** was used as a reference compound, as its biological activity against the PPO receptor has already been elucidated. All proposed ligands (**P4**, **P5**, and **P7**) showed equivalent RMSD results after the first 50 ns of simulation, having a mean RMSD of approximately 0.5 nm. The protein complexed with candidate **P7** exhibited more pronounced fluctuations compared to the others, particularly between 125–150 ns, but achieved consistent stabilization throughout the remainder of the simulation. The complex with the reference **20** showed minor fluctuations over the simulation time, with a mean RMSD of approximately 0.35 nm.

Below, also in Figure 7a, are the results of the RMSD analysis for the coenzyme FAD complexed with the ligands in PPO. For all complexes, FAD demonstrated high stability, with low fluctuations after the first 50 ns of simulation, showing mean RMSDs of approximately 0.15 nm. Finally, Figure 7a displays the RMSF plot of the protein as a function of amino acid residues. For all complexes, the RMSF values remained similar, except for the first and last 20 residues, which showed higher flexibility values. In the residue intervals of 125–150 and 200–250, there was an increase in RMSF for all complexes; the protein complexed with candidate **P7** showed the greatest increase in side‐chain flexibility.

Figure 7b presents the RMSD results for the ligands complexed with PPO. In terms of absolute RMSD fluctuation, ligands **20** and **P4** had RMSDs within the 0 to 0.1 nm range, indicating stability throughout the entire simulation period, with moving averages of approximately 0.075 nm. The normalized histograms reveal that the ligands predominantly maintained RMSDs between 0.05 and 0.1 nm. Ligand **P5** showed evident fluctuations during the first 75 ns of the simulation, subsequently stabilizing at 0.1 nm with low fluctuations, close to its moving average. Despite these initial fluctuations, the histogram shows that candidate **P5** predominantly maintained an RMSD of 0.1 nm. Candidate **P7** showed slight fluctuations during the first 175 ns, with absolute values varying between 0.1 and 0.2 nm. The histogram indicates a predominant RMSD of approximately 0.15 nm, which suggests the ligand's potential for stabilization within the PPO active site.

Following the molecular dynamics simulations, all complexes were submitted to protein‐ligand interaction free energy calculations using MM‐PBSA. These calculations are based on the thermodynamic cycle involving the ligand, the protein, and the complex, in both solvent and gas phases. The details of this calculation methodology are described elsewhere [38].

The energy term for the gas‐phase free energy of the complex (Δ*G*_gas​_) is given by the molecular mechanics (MM) energies, specifically the van der Waals (Δ*E*_vdW_​) and electrostatic (Δ*E*_el​_) contributions. The solvation free energy (Δ*G*_solv​_) depends on the polar solvation parameters, derived from solving the Poisson‐Boltzmann (PB) equation, and the nonpolar solvation energy, derived from the Solvent Accessible Surface Area (SASA). The gmx_MMPBSA program also has the capability to perform entropy calculations (−*T*Δ*S*), which, when summed with the solvation and gas‐phase terms, returns a corrected value for the total interaction energy (Δ*G*_binding​_). The results from the MM‐PBSA calculations can be found in Table 4.

**TABLE 4:** Energetic parameters from the MM‐PBSA calculations (kcal mol^−1^).

Ligand	MM and thermodynamic energies (kcal mol^−1^)					
	∆*E_vdW_*	∆*E_el_*	∆*G_gas_*	∆*G_solv_*	– *T*∆*S*	∆*G_binding_*
**20**	−38.99 ± 7.92	−8.79 ± 1.24	−47.78 ± 9.43	25.58 ± 4.49	5.67 ± 0.05	−16.53 ± 5.69
**P4**	−45.61 ± 2.06	−9.97 ± 2.07	−55.58 ± 2.58	24.24 ± 1.75	4.07 ± 0.04	−27.27 ± 2.50
**P5**	−20.94 ± 3.54	−2.13 ± 0.11	−23.07 ± 3.54	10.16 ± 4.69	4.39 ± 0.05	−8.52 ± 5.88
**P7**	−38.43 ± 2.01	−6.72 ± 2.57	−45.15 ± 2.87	21.91 ± 2.80	3.72 ± 0.16	−19.52 ± 2.65

The van der Waals energy contribution was more evident than the electrostatic energy for all complexes. For ligands **20**, **P4**, and **P7**, the van der Waals energy ranged from approximately −45.6 to −38.4 kcal mol^−1^, with the exception of ligand **P4**, which maintained a value of approximately −21 kcal mol^−1^. The electrostatic energies were relatively high, with emphasis on ligand **P5**, which had the smallest contribution in this aspect. The gas‐phase free energy varied similarly for ligands **20**, **P4**, and **P7** (‐55.6 to ‐45.1 kcal mol^−1^), with ligand **P5** also showing the smallest contribution.

The solvation energy terms account for the solvent effect on complex stabilization and resulted in positive energies for all ligands, with **P4** now being the one that obtained the lowest solvation energy (∼ 10 kcal mol^−1^) compared to the others (22 to 25.6 kcal mol^−1^). The entropic factor showed little variation among the complexes (3.72 to 5.67 kcal mol^−1^), around 2 kcal mol^−1^. The highest entropy is related to the reference ligand **20**.

The interaction energy, which considers all energetic parameters raised thus far, resulted in negative values for all complexes. Candidates **P4** and **P7** revealed more negative interaction energies than the reference ligand **20**. Only candidate **P5** obtained an interaction energy value higher (less negative) than that calculated for the reference.

RMSD is a fundamental parameter for assessing the conformational dynamics of molecules over time. Low values indicate that the conformation remained stable, whereas high values indicate greater variability in conformational states, which can result in lower efficiency [42]. The data obtained from the molecular dynamics simulations revealed conformational stability for all complexes with the QSAR candidates, including the reference ligand **20**, corroborating the results obtained from molecular docking studies. The low fluctuation in the RMSD analyses of the FAD molecule indicates that the presence of the ligands in the active site does not interfere with the coenzyme's stability, preserving the conformational efficiency of the PPO‐FAD complex.

RMSF provides a global assessment of the amino acids' capacity for movement and is directly related to their flexibility [43]. For effective intermolecular interactions to stabilize over time, it is necessary that the residue movement is not so high that interatomic distances vary significantly enough to break the interactions. The RMSF values showed low side‐chain flexibility for the amino acid residues, with only candidate **P7** triggering an increase in flexibility between residues 125–150 and 200–250, which may have impacted the complex's overall stability, as shown in the MM‐PBSA results. In general, regarding RMSD, all ligands behaved analogously to the reference ligand **20** after the first 50 ns of simulation. Few fluctuations were observed, mainly for candidates **P5** and **P7**, but these were still within the range observed for the reference.

MM‐PBSA calculations are widely applied to verify the stability of protein‐ligand systems in molecular modeling protocols for the design of new bioactive compounds [44]. The accuracy of the results compared to experimental data and the low computational cost justify the methodology's application for the rapid assessment of interaction free energies for multiple complexes subjected to molecular dynamics simulations [45]. Table 4 presented the calculated energetic data derived from molecular mechanics and the solution of the Poisson‐Boltzmann equation. The results made it evident that the van der Waals energy contribution outweighed the electrostatic energy for all complexes. This reveals the importance of hydrophobic interactions in stabilization, directly impacting the low free energy values of the complexes in the absence of solvent (gas phase), as shown in the Δ*G_gas​_* column.

The solvation Gibbs free energy term implies the solvent's influence on the stabilization of the complexes. It is expected that the addition of solvent will raise the energy values due to repulsion between the solute and water molecules, this being a determining factor in overall stability. Although ligand **P5** had the smallest energetic impact from solvation (compared to its gas‐phase energy), this candidate was the one with the least negative energy value. Thus, the sum of the gas‐phase and solvation energies corresponds to the Δ*G*_total_, without the entropy correction. Based on this, the ligands, in order of decreasing stability, were **P4**> **20**> **P7**> **P5**.

Besides the energies from the thermodynamic cycle related to complex solvation, another indispensable potential for assessing the stability of the receptor‐ligand interaction is entropy. Interaction entropy, the method used here alongside the PBSA calculations, is an efficient method for calculating the entropic contribution to the binding free energy between proteins and ligands, extracted directly from molecular dynamics simulations at no additional cost [38]. This method improves accuracy and significantly reduces computational cost compared to traditional approaches, making it a valuable tool for molecular interaction studies of various complexes. The variations in entropy were not large from one complex to another, which demonstrates the energetic viability of the QSAR candidates. The addition of the entropic parameter to the calculated free energy terms acts as a correction, increasing the accuracy of the calculations.

After the inclusion of entropy, the interaction free energy between the complexes could be established, indicating the complex with candidate **P4** as the most stable among those analyzed, obtaining a more negative free energy result than the reference compound. Candidate **P7** also proved promising, also with a lower (more negative) energy than the reference used. Only ligand **P5** showed an energy higher (less negative) than **20**, although it was still negative. Therefore, the molecular dynamics studies were able to corroborate the MIA‐QSAR predictions, demonstrating the computational effectiveness of the candidates in the PPO active site. Although a direct correlation between the predicted pIC50 values and the MM‐PBSA binding free energies was not observed, the free energy approach was essential to demonstrate the thermodynamic feasibility of the proposed compounds within the receptor binding site. In this context, MM‐PBSA was employed as a complementary validation to the QSAR results, serving as a form of cross‐validation, rather than as a method intended to quantitatively reproduce the trends in predicted biological activity.

The toxicological profiles of the QSAR‐derived proposals (P4, P5, and P7) were evaluated using the DeepPK platform and compared against the lead compound from the test set (A20) and the commercial reference, sulfentrazone (Table 5). In terms of qualitative classification, all compounds demonstrated a favorable safety profile across multiple endpoints, including AMES mutagenesis, avian toxicity, and biodegradation, all of which were predicted as “Safe” with high confidence (HC). This consistency suggests that the structural modifications implemented to optimize biological activity did not introduce deleterious fragments associated with mutagenicity or environmental persistence. A particularly noteworthy finding is the predicted toxicity toward bees, where sulfentrazone was classified as “Toxic” (LC), whereas P4, P5, P7, and A20 were all predicted as “Safe” (LC). Although these specific predictions carry lower confidence, they indicate a potential ecological advantage for the new proposals, suggesting a reduced environmental footprint concerning pollinator safety compared to the current commercial standard.

**TABLE 5:** DeepPK's tocixity predictions.

Compound	DeepPK toxicity predictions						
	AMES Mutagenesis	Avian	Bee	Biodegradation	Rat (Acute)^a^	Rat (Chronic Oral)^b^	Fathead Minnow^c^
A20	Safe (HC)	Safe (HC)	Safe (LC)	Safe (HC)	2.37	1.00	5.10
P4	Safe (HC)	Safe (HC)	Safe (LC)	Safe (HC)	2.35	0.98	5.07
P5	Safe (HC)	Safe (HC)	Safe (LC)	Safe (HC)	2.43	1.26	4.88
P7	Safe (HC)	Safe (HC)	Safe (LC)	Safe (HC)	2.38	0.98	5.16
Sulfentrazone	Safe (HC)	Safe (HC)	Toxic (LC)	Safe (HC)	2.30	0.91	5.06

^a^ prediction units: ‐log(mol/kg);

^b^ prediction units: log(mg/kg/day);

^c^ prediction units: ‐log10[(mg/L)/(1000*MW)].

The quantitative toxicity predictions should be interpreted with caution due to the different mathematical definitions of each endpoint. For acute rat toxicity and Fathead Minnow (−log scale), higher values correspond to greater toxicity, as they indicate lower doses or concentrations required to produce toxic effects. In contrast, for chronic oral rat toxicity (log of daily dose), higher values indicate lower toxicity, as they reflect a higher tolerated dose.

Based on this interpretation, the proposed compounds (P4, P5, and P7) exhibit acute toxicity values (2.35 ‐ 2.43) that are very close to those of A20 (2.37) and slightly higher than sulfentrazone (2.30), suggesting comparable acute toxicity profiles. For the aquatic endpoint, P7 (5.16) and A20 (5.10) show slightly higher predicted toxicity than sulfentrazone (5.06), while P5 (4.88) presents a more favorable (lower toxicity) profile. Importantly, for chronic toxicity, P5 (1.26) shows a clear improvement over both A20 (1.00) and sulfentrazone (0.91), whereas P4 and P7 (0.98) remain comparable to A20 and still slightly more favorable than the commercial reference.

Overall, when properly accounting for the units and their implications, the proposed compounds can be considered toxicologically equivalent to the reference compounds, with P5 standing out as a promising candidate due to its improved chronic toxicity profile without significant penalties in the other endpoints.

## 4 Conclusions

In this study, robust MIA‐QSAR models were developed to predict the inhibitory activity of protoporphyrinogen oxidase (PPO), based on molecular descriptors representing van der Waals radius and Pauling electronegativity. Model validation showed excellent predictive performance, with high *r* ^2^ values ​​and low RMSE, demonstrating its reliability. Substituent analysis highlighted the importance of the −CF_3_, −F, and −CH_3_ groups for biological activity, aligning with literature results. Based on these predictions, new molecules (**P4**, **P5**, **P7**) with pIC_50_ values ​​greater than 6.50 were proposed, revealing great potential as herbicides. Docking and molecular dynamics simulations corroborated the surface layer of molecules by the PPO active site, and MM‐PBSA calculations indicated that **P4** and **P7** have more negative binding energy than the reference compound, indicating greater stability of the interaction. These compounds show promise as potential candidates for the development of new herbicides, and experimental testing is needed to confirm their biological efficacy and behavior in the field.

## Author Contributions

**Adriana C. de Faria**: investigation, writing – original draft. **Antônio Pedro L. Mesquita**: investigation, writing – original draft. **Elaine F. F. da Cunha**: writing – review and editing, supervision. **Matheus P. Freitas**: funding acquisition, writing – review and editing, supervision, methodology, project administration.

## Conflicts of Interest

The authors declare no conflicts of interest.

## Data Availability

The data that support the findings of this study are available from the corresponding author upon reasonable request.
